# Short-Term Foot and Postural Adaptations During an Industrial Workday: A Workplace-Based Biomechanical Assessment

**DOI:** 10.3390/jfmk10040476

**Published:** 2025-12-09

**Authors:** Alejandro Jesús Almenar-Arasanz, Javier Alfaro-Santafé, Antonio Gómez-Bernal, Jose Luis Perez-Lasierra, Belén Lacárcel-Tejero, José Antonio Villalba-Ruete, Cristina Cimarras-Otal, Juan Rabal-Pelay, Ana Vanessa Bataller-Cervero

**Affiliations:** 1 Department of Physiotherapy, Faculty of Health Sciences, Universidad San Jorge, 50830 Villanueva de Gállego, Spain; aalmenar@usj.es (A.J.A.-A.); joseluisperez@podoactiva.com (J.L.P.-L.); jrabal@usj.es (J.R.-P.); avbataller@usj.es (A.V.B.-C.); 2 Biomechanical Unit, Podoactiva Research & Development Department, Parque Tecnológico Walqa, 22197 Cuarte, Spainantoniogomez@podoactiva.com (A.G.-B.); 3 Department of Podiatry, Faculty of Health Sciences, UManresa, UVic-UCC, 08241 Manresa, Spain; 4 Hospital MAZ, Av. de la Academia Gral. Militar, 74, 50015 Zaragoza, Spain; blacarcel@maz.es; 5 BSH Electrodomésticos España S.A., Av. de la Industria, 49, Santa Isabel, 50016 Zaragoza, Spain; jose-antonio.villalba@bshg.com

**Keywords:** foot posture, plantar pressure, prolonged standing, functional adaptation, center of pressure, occupational biomechanics, arch height, postural fatigue

## Abstract

**Background:** Prolonged standing is common in industrial environments and may induce functional adaptations in the foot and postural system. This study aimed to evaluate short-term changes in foot posture and plantar pressure distribution after a working day in assembly line workers. **Methods:** Forty participants (31 males, 9 females; mean age 44 ± 7 years; BMI 26.1 ± 3.6 kg/m^2^) performed standing tasks during an 8 h shift. Static baropodometric measurements and 3D foot scans were obtained before and after the workday to assess plantar pressure, contact area, and arch height. The Spanish versions of the Cornell Musculoskeletal Discomfort Questionnaire (CMDQ) and the Foot Function Index (FFI) were used to evaluate discomfort and functional status. Paired *t*-tests were applied, and correlations were analyzed (*p* < 0.05). **Results:** Left-foot arch height decreased significantly after the workday (mean change = 0.6 mm; *p* = 0.027). Both mean and peak plantar pressures declined (*p* < 0.001), along with moderate reductions in contact area (*p* ≤ 0.05). The center of pressure shifted mediolaterally, and discomfort was most frequent in the lower back, knees, and feet. A positive correlation was found between arch height reduction and FFI score (r = 0.349; *p* = 0.028). **Conclusions:** Prolonged standing was associated with measurable adaptations in foot posture and plantar pressure, possibly indicating short-term fatigue or compensatory postural adjustments. These results emphasize the importance of assessing plantar load and foot morphology as indicators of potential functional responses to sustained standing and as possible targets for ergonomic and rehabilitation strategies.

## 1. Introduction

Standing posture is one of the most common positions maintained by workers in assembly line industries. Prolonged standing is generally defined as spending more than half of the working day in an upright position [1]. Beyond muscular fatigue, maintaining this posture for extended periods can contribute to mechanical stress and functional alterations that may lead to postural discomfort and work-related musculoskeletal strain [2].

Continuous exposure to static standing produces sustained muscle activation and mechanical loading on the lower limbs and lumbar spine [3,4,5]. This constant demand increases intradiscal and venous pressure, potentially promoting tissue microtrauma, disk degeneration, or venous insufficiency [6,7,8,9,10]. Among assembly line workers, who often have limited opportunities for rest breaks, symptoms such as fatigue, heaviness, and pain in the lower limbs and back are common [11,12]. These effects likely originate from a combination of muscular, vascular, and postural mechanisms, as the absence of dynamic muscle contraction impairs venous return and alters postural stability [13].

The foot plays a crucial role as the primary interface between the body and the support surface, contributing to load absorption and balance regulation. Alterations in foot posture, particularly those affecting the medial longitudinal arch, can modify plantar pressure distribution and contribute to overuse of lower-limb structures [14,15]. Fatigue of the intrinsic and extrinsic foot muscles during prolonged standing may lead to a transient reduction in arch height and increased pronation [16], which can influence plantar pressure patterns and center of pressure (COP) trajectory, affecting postural control efficiency [17].

Several studies have reported that workers exposed to continuous standing experience increased discomfort in the feet, legs, and back, often associated with plantar tissue overload and postural fatigue [18,19]. These adaptations may represent compensatory mechanisms that help workers maintain stability, though at the cost of mechanical efficiency and with the potential for discomfort or fatigue [20].

Prolonged standing has also been associated with alterations in gait mechanics, reduced walking efficiency, and impaired dynamic postural control. Evidence shows that extended periods of upright immobility can modify stride characteristics, increase neuromuscular fatigue, and alter gait variability, suggesting a broader impact on locomotor function beyond static posture alone [21]. These adaptations may persist after the standing exposure and contribute to cumulative biomechanical strain, reinforcing the need to identify early functional changes in the foot and lower limbs under occupational demands.

Understanding short-term adaptations in foot posture and plantar pressure is highly relevant for both ergonomics and movement sciences. Identifying early mechanical changes may contribute to designing preventive and rehabilitative interventions aimed at maintaining postural efficiency and reducing fatigue during standing tasks.

Therefore, the main objective of this study was to analyze the changes in foot posture and plantar pressure distribution over a working day in assembly line workers. A secondary objective was to evaluate the relationship between these biomechanical changes and perceived discomfort in the back and lower limbs, as well as their association with functional foot status.

## 2. Materials and Methods

### 2.1. Subjects

Forty workers (31 males and 9 females; age range: 29–59 years) voluntarily participated in the study. All participants were employees of the assembly line of a Spanish manufacturing company and worked an 8 h morning shift. The inclusion criterion was spending most of the working day in a standing position. Exclusion criteria were pregnancy at the time of the study and the presence of diagnosed lower-limb pathologies or leg-length discrepancy.

All participants were informed about the objectives and procedures of the study and provided written informed consent. The study was approved by the Regional Ethics Committee of Aragón, Spain (C.I. PI20/487) and conducted in accordance with the principles of the Declaration of Helsinki.

### 2.2. Experimental Procedure

Assessments were performed before (PRE) and after (POST) the workday. During both evaluations, static baropodometric recordings and 3D foot scans were obtained to analyze changes in plantar load distribution and foot morphology.

#### 2.2.1. Static Baropodometry

Static plantar pressure measurements were obtained using the Footwork pressure platform (AMCube, Gargas, France), consisting of a pressure-sensitive plate (400 × 400 mm) with 2704 sensors. Data acquisition and analysis were performed using Footwork Pro software (AMCube, Gargas, France), a system previously shown to be valid and reliable [17].

Participants were instructed to stand barefoot in a natural, relaxed position, with their arms at their sides and their gaze directed forward. A single static trial of approximately five seconds was recorded once postural stability was achieved. The parameters analyzed were mean pressure (PMEAN), maximum pressure (PMAX), and contact area (AREA) for both feet. The center of pressure (COP) was also calculated and expressed as a percentage of foot length, where 0% corresponds to the toes and 100% to the calcaneus.

#### 2.2.2. 3D Foot Scanning

Foot morphology was evaluated using a USOL 3D laser foot scanner (Kangzhilai Medical Equipment Co., Guangzhou, China) combined with the elastic membrane system patented by Podoactiva (Podoactiva S.L., Huesca, Spain). This system enables accurate acquisition of foot morphology without soft-tissue deformation [18]. Participants were assessed barefoot in a natural standing posture. The scanner, equipped with dedicated software, captured 3D foot models with a linear precision of 1 mm. Arch height (A_HEIGHT) was calculated for both the right and left foot.

#### 2.2.3. Subjective Measures

Subjective musculoskeletal discomfort and functional foot status were assessed using two validated instruments. The Spanish version of the Cornell Musculoskeletal Discomfort Questionnaire (CMDQ) [19] was used to evaluate discomfort in the back (neck, thoracic, and lumbar regions) and lower limbs (hip, thigh, knee, shank, and foot). The CMDQ quantifies discomfort through three dimensions: frequency, severity, and interference with work productivity. When both sides of the body were assessed, the side reporting greater discomfort was analyzed. Frequency was rated on a four-point scale (“no discomfort” to “several times per day”), severity on a three-point scale (“slightly uncomfortable” to “very uncomfortable”), and interference on a four-point scale (“no interference” to “very intense interference”). For each region, a final CMDQ score was calculated as the product of frequency × severity, providing a quantitative index of discomfort intensity.

Functional foot status was assessed using the Spanish-validated version of the Foot Function Index (FFI) [20,22]. The FFI consists of 23 items divided into three subscales (pain, disability, and activity limitation), each scored from 0 to 10 (0 = no limitation; 10 = maximum limitation). Total scores were summed, divided by 207, and multiplied by 100 to produce a percentage score reflecting overall functional impairment. The FFI was administered only during the pre-work assessment, allowing characterization of baseline foot function prior to the prolonged standing exposure.

### 2.3. Statistical Analysis

Data are presented as mean ± standard deviation (SD) unless otherwise stated. The Kolmogorov–Smirnov (K–S) test was used to assess the normality of data distribution. Paired Student’s *t*-tests were applied to compare PRE- and POST-workday values for both sexes. Pearson’s correlation coefficient was used to analyze associations between normally distributed variables, whereas Spearman’s rank correlation coefficient was used for non-parametric data.

Statistical significance was set at *p* < 0.05. Effect sizes (Cohen’s d) were calculated to quantify the magnitude of differences (<0.2 = small; 0.2–0.8 = moderate; >0.8 = large). All statistical analyses were performed using SPSS Statistics v28.0 (IBM Corp., Armonk, NY, USA).

## 3. Results

### 3.1. Participant Characteristics

Forty assembly line workers (31 males and 9 females) were included in the analysis. The mean age was 44 ± 7 years, and the average BMI was 26.1 ± 3.6 kg/m^2^, corresponding to the overweight range. The mean baseline FFI score was 15.7 ± 20.5%, indicating low-to-moderate levels of functional limitation. Descriptive characteristics for the total sample and by sex are presented in Table 1.

### 3.2. Musculoskeletal Discomfort and Functional Foot Index

According to the CMDQ results, discomfort was most frequently reported in the lower back, knees, and feet. Because these variables were not normally distributed, results are presented as median and interquartile range (Table 2). Women tended to show higher FFI scores than men, reflecting greater baseline functional limitation.

### 3.3. Static Baropodometric Parameters

Static plantar pressure and contact area values before (PRE) and after (POST) the workday are summarized in Table 3. A significant reduction in left-foot arch height was observed (23.2 ± 3.5 mm PRE vs. 22.6 ± 3.3 mm POST; *p* = 0.027; d = 0.31). Among men, this reduction was also significant (*p* = 0.029), whereas no significant change was observed in women.

Mean plantar pressure (PMEAN) decreased significantly in both feet for the total sample (*p* < 0.001), with a larger effect size on the left foot (d = 0.76). Men showed clear reductions in PMEAN bilaterally (*p* < 0.001), whereas women displayed inconsistent changes. Maximum plantar pressure (PMAX) also decreased significantly in both feet for the total sample (*p* < 0.001), with men again showing more pronounced reductions.

Contact area (AREA) decreased significantly in the left foot (*p* = 0.002) and showed a marginal reduction in the right foot (*p* = 0.050). In men, both left and right contact areas decreased significantly, while changes in women were non-significant.

### 3.4. Center of Pressure (COP) Displacement

**Figure 1:**
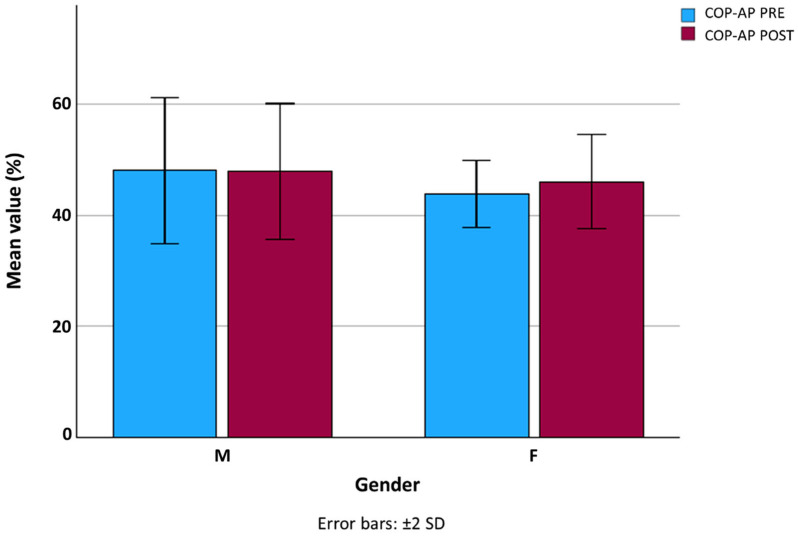
Anterior–posterior center of pressure (COP-AP) displacement in male and female workers before (PRE) and after (POST) the workday. Error bars represent ±2 SD. No significant differences were observed between conditions.

**Figure 2:**
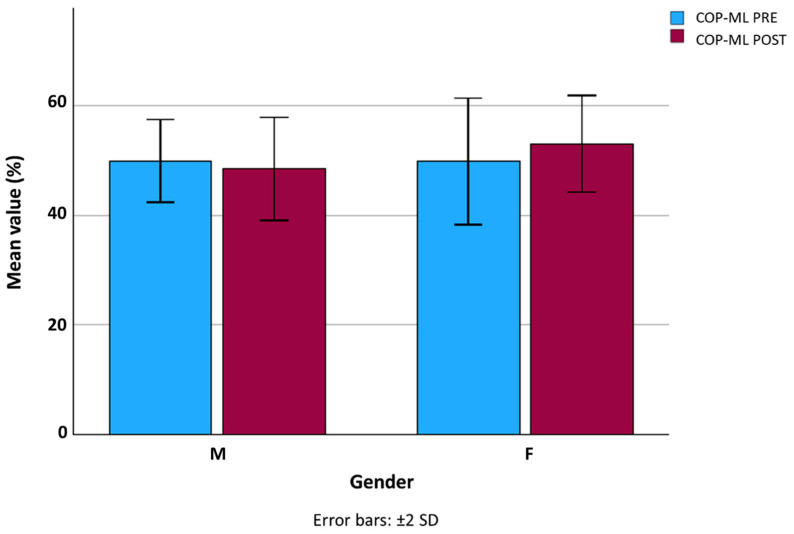
Mediolateral center of pressure (COP-ML) displacement in male and female workers before (PRE) and after (POST) the workday. Error bars represent ±2 SD. Significant differences were found in both men (*p* = 0.030) and women (*p* = 0.041).

No significant differences were found in anterior–posterior COP displacement (COP-AP) between PRE and POST conditions in either sex (Figure 1). In contrast, mediolateral COP displacement (COP-ML) increased significantly after the workday in both men (*p* = 0.030) and women (*p* = 0.041) (Figure 2), which may indicate subtle compensatory adjustments in frontal-plane postural control following prolonged standing.

### 3.5. Correlations

A positive correlation was found between the reduction in arch height and baseline FFI score (r = 0.349; *p* = 0.028), indicating that individuals with greater functional foot limitation experienced larger decreases in arch height over the workday.

## 4. Discussion

This study examined the short-term biomechanical and functional adaptations associated with an 8 h standing workday in assembly line workers. A significant reduction in arch height, particularly in the left foot (from 23.2 to 22.6 mm; *p* = 0.027), may suggest fatigue of the intrinsic and extrinsic foot muscles and a transient lowering of the medial longitudinal arch. This morphological change was accompanied by a reduction in mean static plantar pressure (from 48.9 to 44.2 kPa; *p* < 0.01), possibly indicating compensatory load redistribution that may be related to muscular fatigue. These findings are consistent with those reported by Rabal-Pelay et al. [23], who observed similar modifications in plantar support and discomfort during prolonged standing, emphasizing the multifactorial nature of musculoskeletal complaints in static postures.

The significant reductions observed in PMEAN and PMAX may support the notion that prolonged standing can elicit self-regulatory postural adjustments aimed at minimizing localized overload and maintaining balance. Tarrade et al. demonstrated that customized orthoses can redistribute plantar pressure from the heel to the midfoot, improving postural alignment under sustained load [24]. Similarly, Saadah et al. found that medial arch supports reduce peak pressure and contact area, mitigating fatigue during prolonged standing [25]. Together, these findings suggest that prolonged standing can initiate a combination of morphological and pressure-based adaptations representing short-term postural responses to fatigue.

CMDQ results showed that the lower back, knees, and feet were the regions most frequently affected, with median discomfort scores ranging between 1.0 and 1.5. The mean FFI score (15.7%) reflected mild-to-moderate functional limitation, consistent with prior evidence that prolonged standing induces short-term changes in foot morphology and plantar loading patterns and is associated with increased discomfort among industrial workers [10,23]. Although male participants exhibited more pronounced reductions in plantar pressure, females reported higher functional limitation scores. This discrepancy may be driven by differences in anthropometry, footwear use, and muscle endurance. Coenen et al. previously reported sex-specific musculoskeletal responses to standing work, with women demonstrating greater susceptibility to perceived discomfort despite similar mechanical exposure [8]. Additionally, Parashar et al. found that foot orthotic interventions can improve workplace ergonomics and reduce discomfort in prolonged standing environments [26].

The significant increase in COP-ML displacement observed after the workday may reflect reduced frontal-plane postural control or fatigue of key stabilizing muscles, such as the peroneal and tibialis posterior groups. Prolonged static standing has been shown to diminish the efficiency of lower-limb stabilizers and increase mediolateral sway as a compensatory strategy to maintain balance [13,16]. This pattern suggests that workers may rely on broader, less efficient postural adjustments when fatigue accumulates, even in the absence of major changes in the overall center-of-pressure trajectory.

From an ergonomic perspective, prolonged static standing can trigger biomechanical adjustments that may help preserve postural stability but, if persistent, could contribute to cumulative strain. Several interventions have been shown to attenuate these effects. Lewin and Price reported that custom-made insoles improved plantar load distribution and comfort more effectively than standard insoles [27], while Tardif et al. demonstrated that anti-fatigue mats reduced perceived discomfort by approximately 20%, particularly in the lumbar region [28]. These findings reinforce the importance of preventive strategies targeting both foot mechanics and environmental factors.

Taken together, the present results highlight the relevance of evaluating foot posture and plantar pressure in occupational settings characterized by sustained standing. Implementing ergonomic measures—such as customized orthotic supports, anti-fatigue flooring, scheduled rest breaks, and strengthening programs for the intrinsic foot muscles—may enhance postural efficiency, reduce fatigue, and protect against long-term musculoskeletal strain. However, these findings should be interpreted with caution, as the analysis did not adjust for potential confounders such as sex, BMI, or years of work experience, which may partly influence the observed adaptations.

### 4.1. Strengths and Limitations

A major strength of this study lies in its combination of objective baropodometric and 3D foot-scanning data with validated subjective measures (CMDQ and FFI). Conducting assessments within an actual industrial environment enhanced ecological validity and improved the applicability of the findings to occupational biomechanics. The observed associations between objective parameters and subjective discomfort further support the internal consistency of the results.

However, several limitations should be acknowledged. The statistical analysis did not adjust for potential confounders such as sex, BMI, years of work experience, or habitual standing posture; therefore, residual confounding may be present, and interpretations should be made with caution. The observed arch-height reduction was small (~0.6 mm), and although statistically significant, its clinical relevance remains uncertain and may represent a subtle functional response rather than a structural deformation. Additionally, the combination of reduced arch height and decreased plantar pressure—an atypical pattern compared with the expected increase in loading—may reflect global unloading, muscular relaxation, or alternative postural strategies; further research is needed to clarify these mechanisms. The unequal sex distribution limited statistical power for comparisons between men and women. Finally, post-work discomfort was not reassessed, and the cross-sectional, single-day design precludes conclusions about cumulative or long-term effects of prolonged standing.

### 4.2. Future Directions and Perspectives

Future research should adopt longitudinal or interventional designs to examine how repeated daily exposure to standing influences postural control and plantar loading over time. Incorporating electromyographic and proprioceptive assessments may help clarify the physiological mechanisms underlying the observed functional adaptations. Randomized controlled trials evaluating orthotic devices, anti-fatigue flooring, and structured rest protocols would provide stronger evidence to support ergonomic recommendations.

Moreover, exploring individualized strategies based on foot posture type, muscle endurance, or gender may help optimize preventive outcomes and contribute to improving worker well-being in standing-intensive occupations.

## 5. Conclusions

Prolonged standing during industrial work was associated with measurable biomechanical and functional adaptations in the foot, including reduced arch height, decreased plantar pressure, and subtle postural adjustments. These findings highlight the interdependence between foot function and postural control under static load conditions. Monitoring foot posture and plantar load may serve as a functional biomarker of adaptation or fatigue, offering valuable insight for ergonomic design and rehabilitation strategies. Implementing individualized interventions, such as orthotic supports, anti-fatigue mats, and structured postural variation, may help preserve musculoskeletal health and maintain balance efficiency in occupations that require prolonged standing.

## Figures and Tables

**Table 1:** Baseline characteristics of participants (*n* = 40). Values are expressed as mean ± SD.

Variable	Total (*n* = 40)	Men (*n* = 31)	Women (*n* = 9)
Age (years)	44 ± 7	44 ± 8	43 ± 4
Years working	15 ± 9	16 ± 10	13 ± 5
Height (m)	172.7 ± 7.7	175.0 ± 9.0	163.0 ± 4.9
Weight (kg)	78.3 ± 13.9	80.8 ± 13.6	72.4 ± 7.8
BMI (kg/m^2^)	26.1 ± 3.6	24.9 ± 4.2	22.4 ± 4.5
FFI	15.7 ± 20.5	12.9 ± 16.7	25.2 ± 29.5

Abbreviations: BMI, Body Mass Index; FFI, Foot Function Index. Values by sex are provided for descriptive purposes; no statistical comparisons were performed.

**Table 2:** CMDQ discomfort in back and lower-limb regions and Foot Function Index (FFI) scores in the pre-work assessment. Values are median [IQR].

Region/Variable	Median [IQR]
CMDQ-Neck	1.5 [7]
CMDQ-Upper back	1.5 [3.5]
CMDQ-Lower back	1.5 [3.5]
CMDQ-Hip	0 [1.5]
CMDQ-Thigh	0 [1.5]
CMDQ-Knee	0 [3.5]
CMDQ-Shank	0 [0]
CMDQ-Foot	0 [3.5]
FFI total (%)	7.7 [24.1]

Abbreviations: CMDQ: Cornell Musculoskeletal Discomfort Questionnaire; FFI: Foot Function Index. Values represent the product of frequency × severity for each body region. Data are expressed as median [IQR]. No statistical comparisons were performed; values are presented for descriptive purposes.

**Table 3:** Static baropodometric parameters before (PRE) and after (POST) the workday in assembly line workers. Data are expressed as mean ± SD.

Variable	PRE	POST	*p*-Value	Effect Size (d)
A_HEIGHT_Left (total *n* = 40)	23.2 (3.5)	22.6 (3.3)	0.027 *	0.31
-Men (*n* = 31)	23.8 (3.2)	22.9 (3.2)	0.029 *	0.53
-Women (*n* = 9)	21.0 (3.8)	21.7 (3.8)	0.113	−0.43
A_HEIGHT_Right (total *n* = 40)	23.4 (3.5)	22.9 (3.4)	0.068	0.24
-Men (*n* = 31)	23.9 (3.2)	23.2 (3.2)	<0.001 *	0.35
-Women (*n* = 9)	21.7 (4.0)	22.0 (4.1)	0.332	−0.15
PMEAN_Left (total *n* = 40)	48.9 (7.3)	44.2 (8.7)	<0.001 *	0.76
-Men (*n* = 31)	48.1 (6.8)	41.9 (6.9)	<0.001 *	1.14
-Women (*n* = 9)	51.7 (8.6)	52.1 (9.9)	0.428	−0.71
PMEAN_Right (total *n* = 40)	49.1 (8.9)	43.9 (6.6)	<0.001 *	0.79
-Men (*n* = 31)	47.0 (6.8)	42.8 (5.6)	<0.001 *	0.75
-Women (*n* = 9)	56.3 (11.9)	47.7 (8.5)	0.008 *	0.17
PMAX_Left (total *n* = 40)	123.7 (23.2)	110.9 (24.9)	<0.001 *	0.62
-Men (*n* = 31)	122.8 (22.9)	106.8 (22.8)	<0.001 *	0.68
-Women (*n* = 9)	126.6 (25.6)	124.8 (28.3)	0.404	−0.57
PMAX_Right (total *n* = 40)	124.0 (30.6)	102.4 (20.1)	<0.001 *	0.80
-Men (*n* = 31)	116.7 (21.6)	100.0 (18.1)	<0.001 *	0.39
-Women (*n* = 9)	149.3 (43.9)	110.7 (25.3)	0.009 *	0.17
AREA_Left (total *n* = 40)	75.0 (15.8)	72.1 (15.6)	0.002 *	0.52
-Men (*n* = 31)	77.0 (16.2)	73.7 (15.8)	0.002 *	0.57
-Women (*n* = 9)	68.0 (12.7)	66.5 (14.0)	0.161	−0.33
AREA_Right (total *n* = 40)	75.2 (13.9)	73.1 (12.9)	0.050	0.31
-Men (*n* = 31)	78.2 (12.8)	75.7 (12.4)	0.026 *	0.36
-Women (*n* = 9)	64.6 (12.9)	64.1 (11.2)	0.406	−0.57

Abbreviations: A_HEIGHT = arch height; PMEAN = mean pressure; PMAX = maximum pressure; AREA = contact area. Comparisons between PRE and POST conditions were conducted using paired Student’s *t*-tests. * Statistically significant difference (*p* < 0.05). Cohen’s d was calculated as the effect size (<0.2 = small; 0.2–0.8 = moderate; >0.8 = large).

## Data Availability

The data presented in this study are available upon reasonable request from the corresponding author. Data are not publicly available due to institutional privacy regulations.

## Abbreviations

The following abbreviations are used in this manuscript:

3D	Three-Dimensional
A_HEIGHT	Arch Height
AREA	Contact Area
BMI	Body Mass Index
CMDQ	Cornell Musculoskeletal Discomfort Questionnaire
COP	Center of Pressure
FFI	Foot Function Index
IQR	Interquartile Range
MSD	Musculoskeletal Disorders
PMAX	Maximum Plantar Pressure
PMEAN	Mean Plantar Pressure
